# Effects of Internal and External Factors on Hospital Data Breaches: Quantitative Study

**DOI:** 10.2196/51471

**Published:** 2023-12-21

**Authors:** Diane Dolezel, Brad Beauvais, Paula Stigler Granados, Lawrence Fulton, Clemens Scott Kruse

**Affiliations:** 1 Health Informatics & Information Management Department Texas State University Round Rock, TX United States; 2 School of Health Administration Texas State University San Marcos, TX United States; 3 School of Public Health San Diego State University San Diego, CA United States; 4 Woods College of Advancing Studies Boston College Boston, MA United States

**Keywords:** data breach, security, geospatial, predictive, mobile phone

## Abstract

**Background:**

Health care data breaches are the most rapidly increasing type of cybercrime; however, the predictors of health care data breaches are uncertain.

**Objective:**

This quantitative study aims to develop a predictive model to explain the number of hospital data breaches at the county level.

**Methods:**

This study evaluated data consolidated at the county level from 1032 short-term acute care hospitals. We considered the association between data breach occurrence (a dichotomous variable), predictors based on county demographics, and socioeconomics, average hospital workload, facility type, and average performance on several hospital financial metrics using 3 model types: logistic regression, perceptron, and support vector machine.

**Results:**

The model coefficient performance metrics indicated convergent validity across the 3 model types for all variables except bad debt and the factor level accounting for counties with >20% and up to 40% Hispanic populations, both of which had mixed coefficient directionality. The support vector machine model performed the classification task best based on all metrics (accuracy, precision, recall, *F*1-score). All the 3 models performed the classification task well with directional congruence of weights. From the logistic regression model, the top 5 odds ratios (indicating a higher risk of breach) included inpatient workload, medical center status, pediatric trauma center status, accounts receivable, and the number of outpatient visits, in high to low order. The bottom 5 odds ratios (indicating the lowest odds of experiencing a data breach) occurred for counties with Black populations of >20% and <40%, >80% and <100%, and >40% but <60%, as well as counties with ≤20% Asian or between 80% and 100% Hispanic individuals. Our results are in line with those of other studies that determined that patient workload, facility type, and financial outcomes were associated with the likelihood of health care data breach occurrence.

**Conclusions:**

The results of this study provide a predictive model for health care data breaches that may guide health care managers to reduce the risk of data breaches by raising awareness of the risk factors.

## Introduction

### Background

The health care industry faces the highest number of data breaches among all industries [1]. In the United States, health care data breaches are reported to the Department of Health and Human Services (HHS) Office of Civil Rights (OCR) [2]. An OCR analysis of data breaches reported from October 2009 to December 31, 2020, revealed a substantial increase over the last decade [3]. In 2022, there were 707 breaches reported, a substantial increase from just 18 reported in 2009. Between 2009 and 2015, the loss or theft of medical records and electronic personal health information (electronic protected health information) were the most reported types of data breaches [3].

A survey of health care cybersecurity professionals conducted by the Health Information Management Systems Society in 2022 indicated that 67% of the respondents had major security incidents in 2020 [4]. Ransomware, malicious software that denies users access to their data until they pay a ransom, and phishing emails, fraudulent emails designed to obtain sensitive information, were the predominant causes of these health care security incidents [4-6]. During 2021, the data in >45 million patient medical records were affected by cyber threat actors, which was a large increase from 34 million records affected in 2020 [7,8]. A major concern is that many cyber attackers were able to roam around the breached systems for extended periods. In 1 case, they exploited the breached system for >7 years before being detected [7]. This raises the distinct possibility that many health care systems harbor undetected breaches.

The Health Information Technology for Economic and Clinical Health Act Breach Notification Rule requires health care organizations covered by the Health Insurance Portability and Accountability Act (HIPAA), called covered entities, to report breaches to the HHS [9]. For data breaches affecting >500 individuals, the breach details are posted publicly on the OCR website [10]. The OCR imposes heavy monetary penalties on organizations that have experienced data breaches. However, the number of health care breach penalties continues to increase. In 2022, the OCR imposed a record number (707) of penalties, surpassing any other year since 2006, when they were granted authority to enforce HIPAA rule compliance through financial penalties [11].

### Economic Impacts

Data breaches are costly for health care facilities. The third largest penalty ever levied was to Advocate Health Care for US $5.5 million for the theft of desktop computers containing data that affected >4 million patients [12]. In 2023, Banner Health, with 30 hospitals and multiple specialized health facilities, paid US $1.25 million for multiple risk management issues such as a lack of technical safeguards for verification of identity to access protected health information. In 2017, the Children’s Medical Center of Dallas paid US $3.2 million for the loss of a mobile device with the electronic records of 3800 patients [13]. In addition to HIPAA-compliance penalties, class action lawsuits have been brought against facilities by health care consumers whose patient or financial data were compromised [11].

Although cyberattacks at smaller facilities are not as common, their number is increasing. In 2022, more cyberattacks occurred at small hospitals, clinics, and technology companies with smaller budgets, smaller staff, and cyberdefense deficits [14]. As an illustration, 12% of the health care data breaches in the first half of 2022 occurred in physician groups and health care services, and 14% occurred in health care service and supply companies [14]. This is concerning because the economic impact of health care data breach penalties and the loss of consumer trust threatens to put these small health care businesses out of business [15].

### Risk Assessment

A HIPAA risk assessment considers the level of threats and vulnerabilities to protect health information and assesses an organization’s current security measures. Cybersecurity attacks on health care data are risk management issues because they are associated with disruptions in business operations, adverse impacts on the organization’s reputation, exposure or loss of sensitive patient data, risk of litigation, potential loss of revenue, financial penalties, and possible negative consequences for the patients [16,17]. For example, a Ponemon Institute 2022 report noted an increase in patients transferred to other facilities and an increase in mortality rates as harmful outcomes of data breaches [18]. Unfortunately, as of 2019, failure to perform a thorough risk analysis assessment is one of the most frequently cited HIPAA security rule violations [19]. Another HIPAA OCR audit report from 2016 to 2017 found that 86% of the covered entities failed to conduct an in-depth risk analysis and 94% of all covered entities did not meet risk management expectations [20].

The National Institute of Standards and Technology Framework for Improving Critical Infrastructure Cybersecurity suggests that organizations should work diligently to identify and address cyber risks to mitigate the likelihood of experiencing a data breach [21]. A productive proactive risk management approach would be to predict the likelihood of a health care data breach before it occurs. However, there is limited direct comparable research on internal and external factors such as hospital characteristics, economics, demography, and geographical factors associated with experiencing health care data breaches.

### Factors Associated With Risk

#### Internal

A few studies considered the internal factors of health care data breaches. Kamoun and Nicho [22] proposed a medical data breach causation chain that included organizational influence, inadequate security defenses, and unsafe data handling adapted from Reason’s Swiss cheese model. Zafar et al [23] suggested that organizational maturity may be associated with an increased number of data breaches. Angst et al [24] determined that older, smaller, for-profit, nonacademic, and faith-based hospitals were more likely to experience a data breach.

A related 2018 study adapted the Kamoun and Nicho [22] model to assess HHS OCR breach data and Health Information Management Systems Society Analytics data from >6000 health care facilities in the United States on the constructs level of data exposure, facility security, and organizational characteristics [25]. The results of this 2018 study indicated that the number of staffed hospital beds, number of outpatient visits, number of intensive care beds, number of surgical operations, total operating expenses, and the year the facility was opened were associated with the likelihood of experiencing a health care data breach. Higher use levels for electronic medical records (EMRs), percentage of physicians using Computerized Provider Order Entry (CPOE) systems, barcode use by laboratories, and number of births were also associated with higher breach risks [25]. EMRs are generally local to a physician’s clinic practice, whereas electronic health records (EHRs) are intended for maximum interconnectivity outside the health care facility. CPOEs are systems for entering the medication, laboratory, and radiology orders.

A systematic review of antecedents and consequences of data breaches by Schlackl et al [26] explored antecedent categories for managerial (security investments and policies, business partnerships, and IT governance), technological (security technologies and system auditing), organizational (size and industry), and regulatory factors (US data breach notification laws and regulatory enforcement). They reported that larger organizations and organizations with EHRs reported more data breaches, whereas organizations that conducted financial audits reported fewer data breaches.

Williams [27] explained how health care organizations are tackling the “bring your own device” trend. Robust wireless service is an expectation from providers and patients. Clinicians feel that smartphones and tablets are indispensable assets to their workflow [28]. Handheld computing devices are used by many providers such as nurses for workflow tasks such as decision support, and mobile apps are in high demand [29,30]. Since the COVID-19 pandemic, telemedicine has greatly increased, and it will most likely continue [31].

Hospitals use industry-standard practices to ensure the security of data within the firewall of the hospital. Incident to care, however, patients, their families, and vendors also introduce personal devices into the hospital network. Security specialists segment medical devices and monitor consumer devices [32]. Hospitals often create 1 wireless network that uses Wi-Fi Protected Access, version 2–Enterprise security and create a demilitarized zone for a guest network that does not require authentication. Although patients, their families, and vendors use the guest network, their personally owned devices create an inherent vulnerability to the network. The demilitarized zone mitigates this risk. The use of personally owned devices in the hospital creates an intersection of cybersecurity threats from the general population and cybersecurity in the hospital.

#### External

There is scarce research on health care data breaches at the county level that considers external factors such as demography and geography, which creates a gap in the literature. Regarding demography, security studies consistently provide evidence that lax security practices of end users are associated with data breaches [33-36]. Moreover, the ubiquitous provisioning of health care services over the internet is concerning because breaches invoked by remote users are costlier, averaging approximately US $1 million more than the average costs for breaches not associated with remote access [37]. However, few studies considered the association between population demographics and health care data breaches.

Chua et al [38] explored the effects of age, gender, ethnicity, education level, work experience, and industry on individuals’ awareness of organizational security practices. The results indicated that age, working industry (financial services, IT, health care, insurance, and others), and education level were predictors of increased awareness of and compliance with organizational security practices, with age accounting for 55% of the security awareness score [38].

#### Geography

Geospatial effects on health care breaches have been explored for states and regions by a few researchers. Raghupathiet al [39] analyzed state-level health care data breaches reported to the HHS OCR. Their results indicated that hacking and server breaches affected most individuals. They also revealed that the number of breaches, the number of affected individuals, and the types of breaches varied by state [39]. California, Texas, New York, and Illinois reported the most breaches, but breaches on the East Coast affected more individuals. The type of breach varied by state, with California experiencing the most breaches from the theft of computers containing data [39].

A related 2019 study analyzed HHS OCR breach data from 2009 to 2018, revealing that hacking or IT incidents and server breaches were the most significant determinants of the number of affected individuals [40]. Although geographic region was not a significant predictor of having a data breach, descriptive statistics indicated that the southern region had the most individuals affected, and the Midwest region had the fewest number of individuals affected [40].

### Study Hypotheses

A consideration of the previous empirical literature guided this study. Our review highlighted the scarcity of studies focused on the external environment and its association with risks of health care data breaches at the county level in the United States, which is the main gap in the literature we are seeking to fill with this study. In addition, we aim to add to the limited research on the internal factors associated with risks of health care data breaches at the county level. Ultimately, we intend to develop a predictive model to guide hospital and health care managers to understand the factors that put their facilities at risk for health care data breaches.

Existing literature indicated that internal factors, such as patient workload, facility type, and financial and economic outcomes, and external factors, such as demographics and geography, were predictive of data breach occurrences. Therefore, we hypothesize as follows:

- Hypothesis 1: internal factors of hospitals will be associated with risks of health care data breaches at the county level in the United States.
- Hypothesis 2: external factors of hospitals will be associated with risks of health care data breaches at the county level in the United States.

## Methods

All analyses and data are available on the web [41].

### Data

The Definitive Healthcare data set provided the hospital-related data and the hospital breach information for this study [42]. The Definitive Healthcare database combines US hospital data sources such as Medicare Cost Reports, commercial billing claims data, Medicare Standard Analytics Files, Centers for Medicare and Medicaid Services Hospital Compare, and other data elements. The US Census Bureau served as the data source for population, demographic, and geographic variables [43]. The Bureau of Labor Statistics provided unemployment data [44]. All data were joined at the county level for predictions, and the cross-sectional year for the analysis was 2022.

### Software

All analyses were performed in R statistical software (R Core Team) and Python (version 3.1X; Python Software Foundation) via the package *reticulate* using the R Studio Integrated Development Environment (Rstudio Team). Specific packages (libraries) invoked are available on the web [45,46].

### Sample and Unit of Analysis

After merging the Definitive Healthcare and Census Bureau data sets, there were 1032 counties (unit of analysis) with hospital information available out of 3233 counties in the Census Bureau’s shapefile. Some counties had no reported breaches, so zeros replaced the Not Applicable coded as “NA.” All remaining data were complete, resulting in a final sample size of n=1032.

### Dependent Variable

The initial dependent variable (DV) counted the number of breaches in each county; however, there were only a few counties (n=74) in which >1 breach was reported during 2022. Thus, the variable was collapsed on the set {0=no breach reported, 1=breach reported}.

### Independent Variables

The independent variables were separated into 5 groups of variables based on category: demographic, workload, financial, facility type, and economic indicators. Operational definitions of the variables by type are provided in the following subsections.

#### Demographic Variables

Demographic variables included the proportion of American Indian, Asian, Black, and Hispanic populations and those aged >65 years. Each of these variables was measured on the domain of {0,1} and was acquired from the Census Bureau.

#### Workload Variables

Three workload variables were included in the model: 1 for inpatient workload, 1 for outpatient workload, and 1 for average length of stay (ALOS). The outpatient workload was straightforward and measured as the number of annual outpatient visits (integer). An outpatient visit is defined as contact with a health professional such as a physician (both generalists and specialists), nurse, midwife, and dentist outside of admission to any health care facility and without occupying a hospital bed for any length of time. These visits were reported in thousands and averaged at the county level.

For inpatient workload, a feature-engineered measure was developed (denoted “BedFreqSev” in the data set). This feature was designed to estimate inpatient workload and severity as the number of acute beds multiplied by the bed percent utilization (resulting in the average daily acute beds demanded) multiplied by the average case mix index (CMI). An acute bed is defined as a bed allocated to patients with the need for obstetrics (ie, managed labor), injury treatment, surgery, symptom relief or management of nonmental illness, and diagnostic or therapeutic procedures, whereas bed use is the average bed use percentage measured by dividing the total number of patient days (excluding nursery days) by the number of bed days available. Multiplying the 2 terms together generates the total daily demand for acute beds. CMI is a measure of patient severity based on Medicare inpatient claims for the most recent calendar year and is used to determine the allocation of resources to care for a hospital’s patients. Payment weights are assigned to each diagnostic-related group based on the average resources used to treat Medicare patients in that diagnostic-related group and are calculated by summing the Medicare Severity-Diagnosis Related Group weight for each discharge and dividing the total by the number of Medicare claims. CMI is averaged at the county level. Multiplying the total daily demand by CMI provides a measure of severity-weighted inpatient demand.

The last measure used for workload was ALOS. ALOS is the average number of days that patients spend in the hospital, measured by dividing the total number of patient days by the number of Medicare claims (averaged by county). This variable reflects the per-patient inpatient workload and is averaged across the county.

#### Financial Variables

Operating profit margin, capital expenditures, operating income, accounts receivable (AR), and bad debt were the financial variables in the study. Operating profit margin is the amount by which revenue exceeds costs and is calculated as net patient revenue minus total operating expenses divided by the net patient revenue. This variable from Definitive Healthcare was reported as a percentage and averaged at the county level. Capital expenditures are the sum of the cost of capital assets acquired by the purchase of land, land improvements, buildings, fixtures, building improvements, fixed equipment, movable equipment, and health information technology– designated assets. These expenditures were initially averaged at the county level and are reported in millions. Operating income is the net patient revenue minus the total operating expense averaged at the county level and is reported in millions. AR includes all unpaid inpatient and outpatient billings (especially direct patients for deductibles, coinsurance, and other chargeable items, if not included elsewhere in the Medicare Cost Report). AR is averaged at the county level and is reported in millions. Finally, bad debt for a facility represents the total amount of bad debt for the entire hospital complex as written off during the Medicare Cost Report reporting period on balances owed by patients, regardless of the date of service. Bad debt is averaged at the county level and is reported in millions.

#### Facility Type Variables

Many counties had specialty hospitals within their borders including pediatric trauma and medical centers. Indicator variables were used to indicate their presence or absence within a county.

#### Economic Variables

Both the unemployment and poverty variables served as controls at the county level. The unemployment rate was the county’s unemployment percentage for the year 2019, as reported by the Census Bureau. The poverty rate (also defined and provided by the Census Bureau) was the percentage of the county’s population determined to live below the official US poverty level.

### Training and Test Set Split

For analysis of model performance, data were split into training and test sets (825/1032, 79.94% and 207/1032, 20.06%, respectively) using a pseudorandom number seed to support replication of the analysis. This split occurred before any transformation to avoid information leakage from the training set to the test set.

### Transformations-Discretization

#### Overview

Some variables were both nonnormal and resistant to transformation but benefited from discretization. These variables included the proportion of American Indian, Asian, Black, and Hispanic populations as well as capital expenditures. These variables were discretized (converted to qualitative variables) by generating factor levels at each quintile {0%≤x<20%, 20%≤x<40%, 40%≤x<60%, 60%≤x<80%, 80%≤x<100%}. These quintiles were calculated on the *training* set variables only and then used to dichotomize both the training and test set variables based on the appropriate values. Information from the test set therefore remained unknown and unused. For all variables, the reference level was set to the most commonly occurring factor level.

#### Power Transformations

Other variables were nonnormal but made reasonably normal by transformations that did not require the use of statistics generated from the overall data, thereby avoiding data leakage and contamination of the test set. Logarithmic transformations of outpatient visits, the engineered inpatient workload-severity variable (+0.01), and bad debt resulted in largely normal distributions. A square root transformation of the poverty variable also generated a seemingly Gaussian distribution. Finally, an inverse square root transformation of unemployment resulted in a more normal appearance. Although the normality of each variable (or multivariate normality) is not required, these simple and logical transformations empirically improved model performance. The shift of +0.01 for the engineered variable ensured that it was positive definitive.

#### Scaling

Some methods including regularized logistic regression and perceptron are not scale invariant. Therefore, larger-magnitude variables may unnecessarily take on more importance in the modeling process. To avoid scaling issues, all variables were Z-normalized using means and SDs from the training set data to avoid information leakage. Equations 1 and 2 illustrate these transformations.



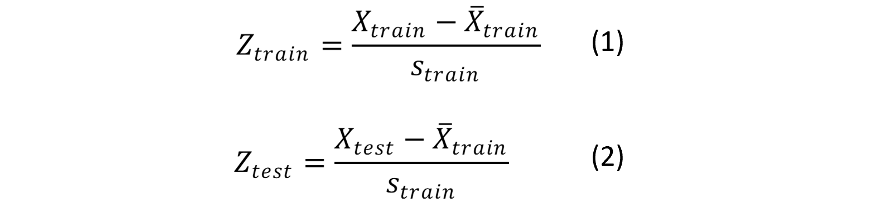



In equations 1 and 2, the means from the training data variables are subtracted from the training and test set variable observations and subsequently divided by the SD of the training set variables. Using only the training set statistics prevents information leakage.

#### Balancing the Training Set

In the entire data set, only 22.67% (234/1032) of the observations involved breaches. When the data were split into training and test sets, only 189 (22.9%) of the 825 observations were breaches. To improve the classification performance, the majority-weighted minority oversampling technique from the *mwmote* library in the R *imbalance* package was applied to generate 500 additional but synthetic breach cases for the training data only [47]. This technique assigns weights for the hard-to-learn minority class samples based on their Euclidean distance from the nearest majority class. Synthetic samples are then generated from these weighted minority class samples via clustering. The test set was left imbalanced and unadulterated [47].

### Descriptive Analysis

The univariate, bivariate, multivariate, and spatial descriptive analyses provided researchers with an understanding of breaches and their locations. Demographic analysis was descriptive only, as there were insufficient counties to build robust geospatial statistical models.

### Models

#### Logistic Regression

All inferential statistical analyses were conducted at the county level. Logistic regression models (regularized and otherwise) were used to estimate the presence of a breach as a function of the other variables. Logistic regression is well suited for 2-class classification where linear models necessarily fail the homoscedasticity assumption as the variance of each Bernoulli-distributed observation 
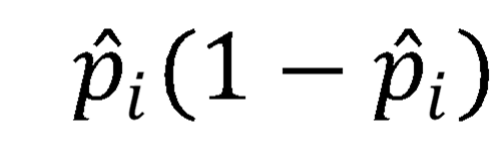
 necessarily changes based on the probability estimate (
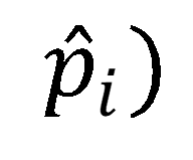
) of the dichotomous DV [48]. For example, a probability estimate of 0.1 yields a variance of 0.09, whereas a probability estimate of 0.5 yields a variance of 0.25, instant heteroskedasticity. Furthermore, the Gaussian link function allows for values between minus infinity and infinity, yet the DVs exist on {0,1}.

Modeling the probability of having a breach for observation *i* as a logistic cumulative distribution function that exists on the domain {0,1} with a heavier presence near the extremes results in an estimated probability of 
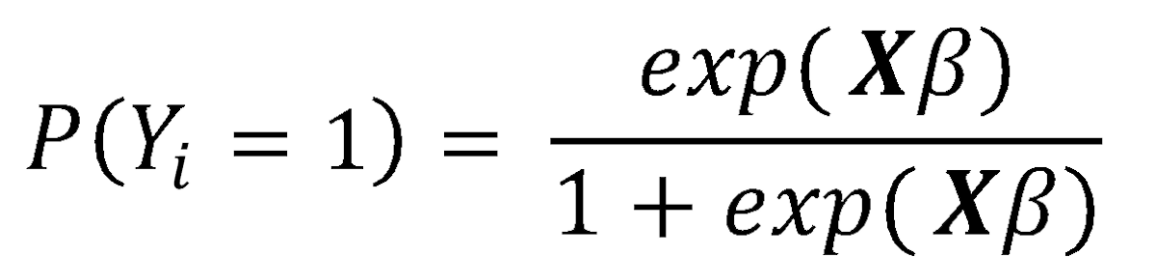
 with the complement 
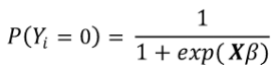
 (note: X is the independent variable matrix augmented by a leading column of 1’s to account for the intercept or bias). The ratio 
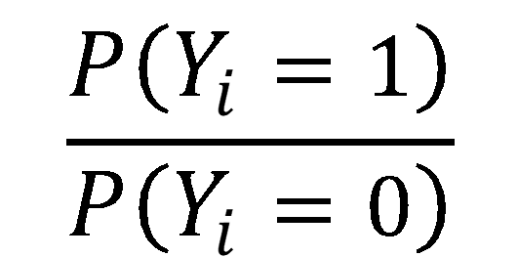
 is the well-known odds ratio (OR) and is simplified to *OR* = exp(Xβ). Taking the log of both sides returns the linear in parameters equation, log (*OR*) = Xβ.

Estimating the β vector is often performed using maximum likelihood estimation. Although logistic regression avoids the assumptions of homoscedasticity (embracing the Bernoulli model), some assumptions do exist, including the linearity of the log odds for continuous variables, absence of collinearity, absence of extreme outliers, and independence of observations [49]. All assumptions were investigated.

#### Perceptron

**Figure 1:**
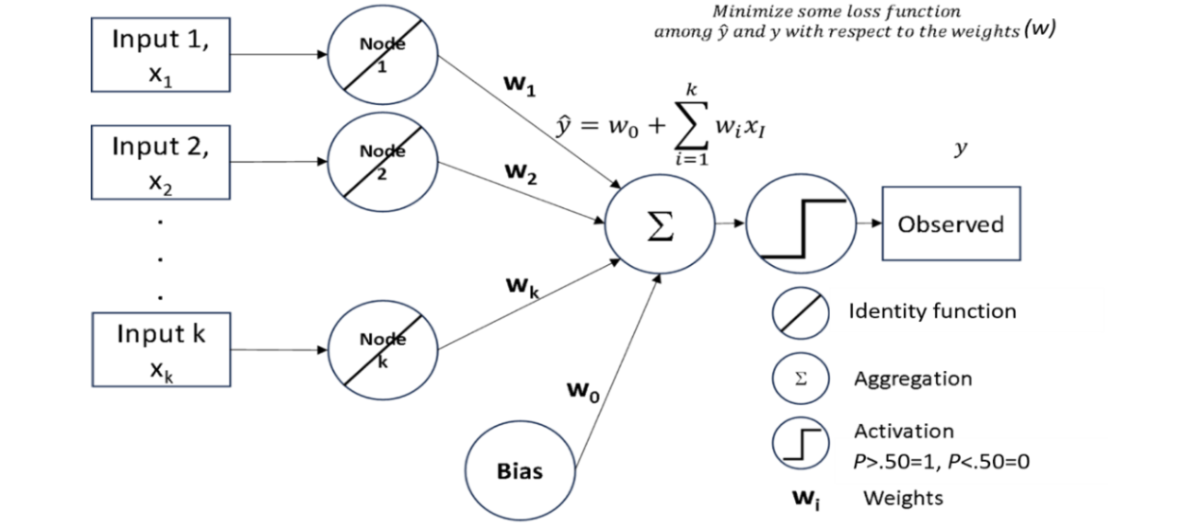
A perceptron model.

Perceptron models using the traditional additive function were also built to investigate classification performance. These neural networks use a bias term coupled with neurons equal to the number of inputs and then aggregate them to produce an estimate of the DV [50]. The advantage of perceptrons is that (given properly scaled data) they are assumption less and produce coefficient estimates for each input variable–interpretable machine learning. The weights between the inputs and neurons may be tuned via nonlinear optimization. If the activation function for a perceptron is changed to a logistic function, then the output is identical to that of logistic regression. The value of the logistic assumption can be evaluated using the traditional aggregation function. Figure 1 illustrates a typical perceptron network.

In Figure 1, inputs are pushed directly to neurons (nodes or the circle with the / identity function indicator) and weighted for aggregation (circle with the + sign). The aggregated estimate can be compared with the observed data, and then the weights can be adjusted to reduce any error loss function. The result of the perceptron is a set of weights (w) that are coefficients indicating the direction and magnitude associated with each separate input, a convenient truth for comparing with logistic regression models.

#### Linear Support Vector Machine

A linear support vector machine (SVM) was also used to generate classification results. SVM maximizes the width of the gap (maximum margin) between the parallel linear bifurcations of the 2 categories. As a statistical learning optimization problem, it is not bound by traditional statistical assumptions other than the idea that data are independent and identically distributed. Maximizing the margin is a linear optimization problem [51]. Letting *M* be the margin, then the optimization is equations 3, 4, and 5:



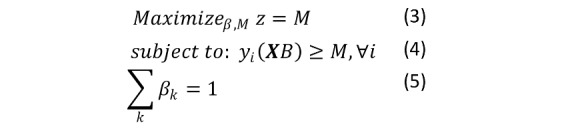



This equation maximizes the margin (big *M* maximization in traditional optimization) while requiring that the hyperplane separation is larger than *M* for both *y_i_* = {−1, 1} and ensuring that the standardization of the coefficients to ensure that each observation is on the proper side of the hyperplane [52].

#### Regularization (Shrinkage Methods)

All models were investigated with and without regularization. Regularization, a form of shrinkage, adds a penalty function to drive variables out of the model, resulting in a more parsimonious expression of the prediction equation. Regularization techniques such as ridge regression, lasso regression, and elastic net penalize the inclusion of additional variables. Ridge regression minimizes the residual sum of squares with respect to β as in ordinary least squares but includes an L2-norm penalty function in the objective function minimization: 
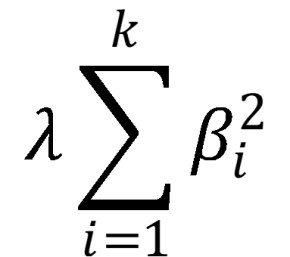
. The penalty function attempts to reduce many of the parameter estimates (β) to near 0 and is tuned during cross-validation. When λ = 0, ridge regression is the same as ordinary least squares. Although ridge regression reduces some parameter estimates as λ increases, all estimates (no matter how small) remain part of the model (unless λ = ∞), unlike lasso regularization. Lasso regularization also seeks to minimize the residual sum of squares; however, the penalty function is an L1-norm penalty, 
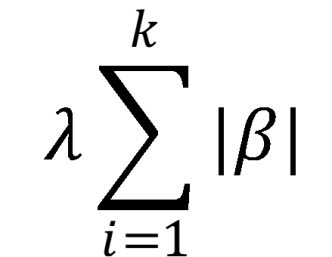
. The advantage of lasso is that this penalty forces some of the parameter estimates to exactly 0 rather than near 0. Again, the value of λ is set using cross-validation. Elasticnet regularization includes a linear combination of both ridge and lasso penalty functions: 
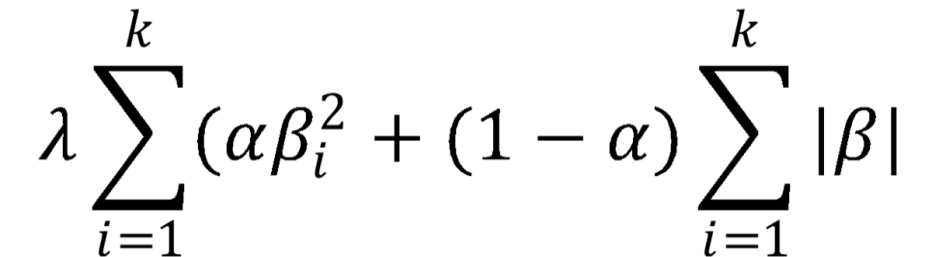
. The value of the mixture hyperparameter can also be tuned via cross-validation [53].

The models presented in the results include M1—the full logistic regression model (all assumptions evaluated), M2—the perceptron model (optimal regularization), and M3—the linear SVM (optimal regularization). These 3 models were compared in terms of coefficient stability and prediction performance.

### Metrics

Models were built on the augmented training set (ie, the training set including synthetically generated observations using *mwmote*) and forecasted on the pristine test set to evaluate performance. The models were built sequentially by block. Accuracy, precision (positive predictive value), recall (sensitivity), specificity, and other metrics including the *F*1-score (harmonic mean of precision and recall) and pseudo-*R^2^*, 
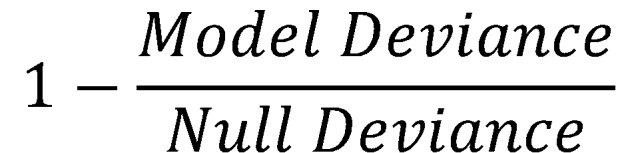
, were compared to evaluate model performances.

### Ethical Considerations

Ethical review and approval were not needed for this study because the study data were publicly available data on hospitals, not humans. Our main data source was the Definitive Healthcare data set which provided comprehensive data extracted from publicly available information, including from federal, state, and other regulatory agencies, in addition to licensed data from other companies. Other data sources were the US Census and the Bureau of Labor Statistics provided unemployment data.

## Results

### Descriptive Statistics Results

Table 1 provides descriptive statistics at the county level of analysis. Means of proportions are reported as percentages. The percentage of counties reporting breaches was 22.7% (SD 41.9%). The mean proportion of American Indian, Asian, Black, and Hispanic populations as well as those aged >65 years per county was <1% (SD 3%), 2.6% (SD 3.9%), 10.3% (SD 13.3%), 10.9% (SD 13.5%), and 17.9% (SD 4.2%), respectively. The percentage of unemployed people in these counties was 3.86% (SD 1.222%), whereas the mean poverty was 13.98% (SD 5.183%).

The average number of outpatient visits was 186,414 (SD 233,411), and the severity-weighted acute bed demand was 619 (median 193, SD 1299.7). Counties had on average only 52% bed utilization with 587 acute beds and a CMI of 1.57. The ALOS for inpatient care was 4.54 (median 4.57, SD 0.895) days. The average profit margin for hospitals operating in any county was −0.01% (SD 0.169%), indicating that, on average, they were operating at a loss. Capital expenditures averaged US $58.63 million (SD $56.0 million), whereas the mean operating income was negative (−US $3.26 million, SD 97.5 million). The average hospital in each county had US $151.11 million in AR (SD $268.3 million) and US $23.37 million in bad debt (SD $34.5 million).

Just 5.04% (52/1032) of the counties had pediatric trauma centers, whereas 10.95% (113/1032) had medical centers, which are large facilities providing medical and surgical care that are associated with a medical school [54].

**Figure 2:**
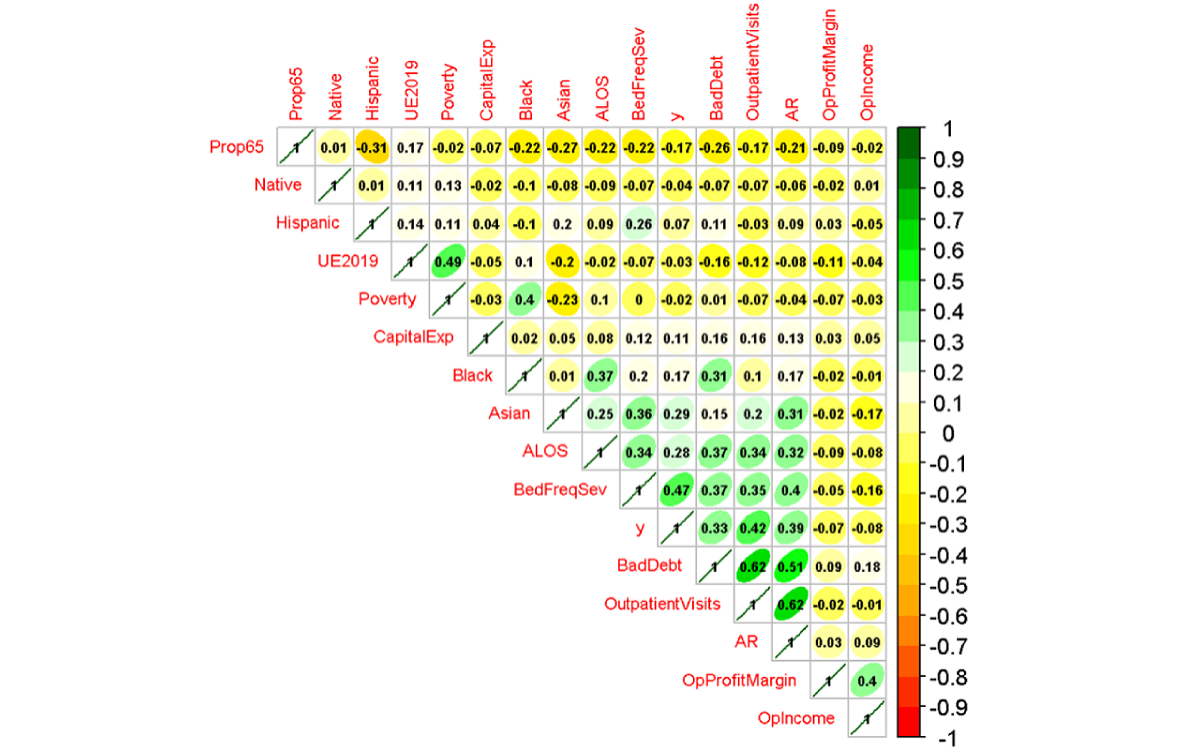
Hierarchically clustered correlogram. AcuteBeds: acute beds; ALOS: average length of stay; AR: accounts receivable; BadDebt: bad debt; BedFreqSev: inpatient workload and severity; BedUtil: bed use; CapitalExp: capital expenditures; CMI: case mix index; Native: percentage of American Indian; OpProfitMargin: operating profit margin; OutpatientVisits: outpatient visits; Prop65: percentage of those aged >65 years; UE2019: unemployment percentage in 2022; y: breaches (dichotomous).

Figure 2 shows a hierarchically clustered correlogram of the relationships among all variables before transformations and scaling. This figure illustrates that the highest magnitude correlations are among bad debt and AR (0.62) as well as outpatient visits and operating profit margin (0.62), findings that are not unexpected. Poverty and unemployment also show some correlation. The correlogram suggested the possibility of collinearity; therefore, a complete variance inflation factor (VIF) investigation was conducted for the logistic regression model. Furthermore, the correlogram illustrates that the DV (“y”) exhibited mild point biserial correlation with ALOS, inpatient workload or severity (“BedFreqSev”), and the proportion of Blacks in the counties (“Black”).

**Figure 3:**
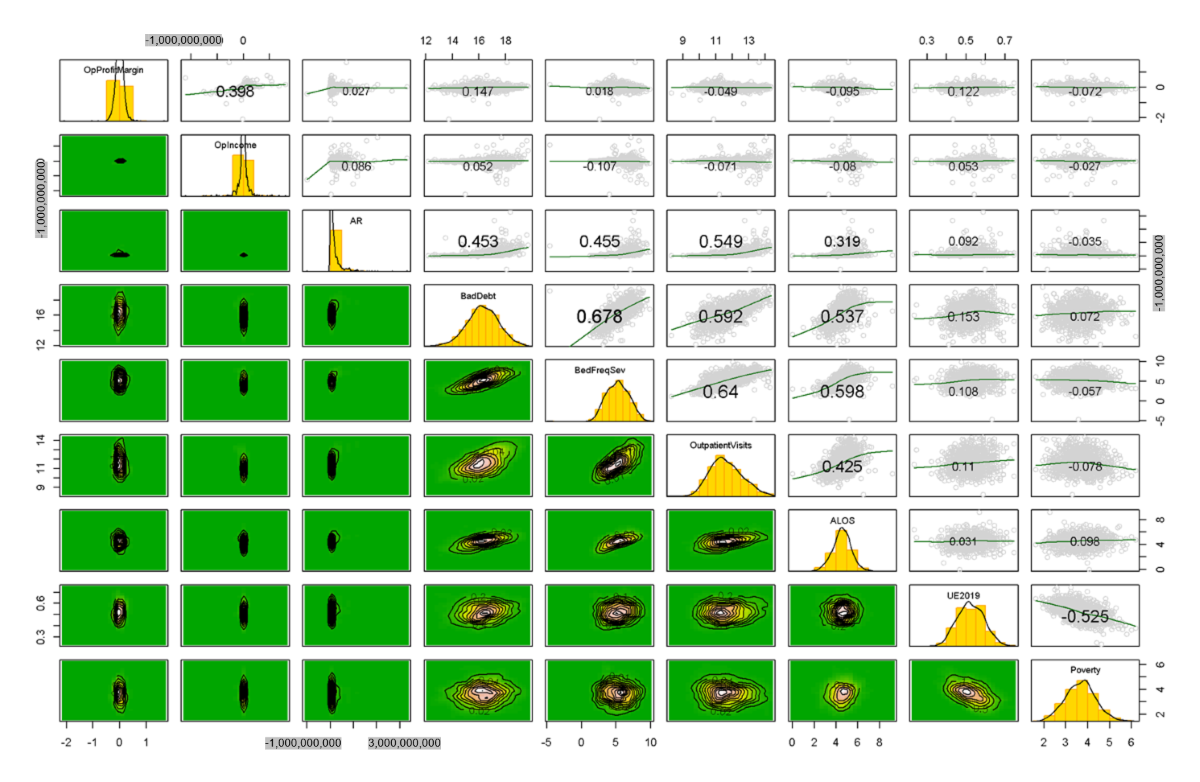
Pairs plot of the nondiscretized variables, transformed variables before Z-scaling. ALOS: average length of stay; AR: accounts receivable; BedFreqSev: bed frequency and severity; OPIncome: operating income; OPProfitMargin: operating profit margin; Poverty: poverty by county; UE219: unemployment in 2019.

As previously discussed, the initial pairs plot of the quantitative variables demonstrated significant nonnormality in many variables. Before transformations, data were split into training and test sets to avoid information leakage. Discretization of the power-transform–resistant variables was conducted using quantiles from the training set applied to the test set. Obvious transformations such as logarithms and square root transformations supported by Box-Cox analysis were then conducted on the remaining quantitative variables, resulting in a largely normal appearance of distributions, as illustrated in Figure 3, a pairs plot. After discretization and transformations, data were Z-scaled using the means and SDs from the training set and applied to the test set, as discussed previously. Then, the training set was augmented with synthetic data (*mvmote*). Both the training and test sets were then ready for modeling.

In Figure 3, the histograms are depicted on the diagonal with bivariate boxplots in the lower triangular matrix. In the upper triangular matrix, pairs plots with loess curves and correlation coefficients are depicted (text size indicates the correlation magnitude).

**Table 1:** County-level descriptive statistics (N=1032).

	Values, mean (SD)	Values, median (IQR)	Range (minimum-maximum)
Breaches (Bernoulli random variable)	0.227 (0.419)	0.000 (0 to 0)	0.000-1.000
American Indian	0.009 (0.030)	0.003 (0.002 to 0.005)	0.000-0.392
Asian	0.026 (0.039)	0.013 (0.007 to 0.030)	0.000-0.431
Black	0.103 (0.133)	0.048 (0.015 to 0.132)	0.000-0.774
Hispanic	0.109 (0.135)	0.059 (0.031 to 0.127)	0.002-0.955
Proportion aged >65 y	0.179 (0.042)	0.175 (0.15 to 0.199)	0.079-0.408
Inpatient workload	616.926 (1299.735)	192.567 (57.656 to 608.508)	0.000-19,129.733
Outpatient visits	186,413.545 (233,411.477)	100,676.250 (55132 to 215685.25)	3904.000-1,713,803.000
Average length of stay	4.539 (0.895)	4.567 (4 to 5.1)	0.000-9.200
Operating profit margin	−0.008 (0.169)	−0.010 (–0.085 to 0.073)	−2.091-1.654
Capital expenditures (US $)	58,634,325 (560,360.299)	10,315,109 (3,573,528.500 to 28,170,072.625)	–213,031,958-15,898,581,598
Operating income (US $)	–3,257,404 (97,489,175)	–1,388,767 (–17,422,500 to 16,088,302.500)	–1,109,645,967-802,473.902
Accounts receivable (US $)	151,104.585 (268,250.395)	61,440,467 (25,232,239.500 to 159,857,281.750)	–1,012,900,560-3,306,532,176
Bad debt (US $)	23,366,222 (34,484,395)	11,760,138 (5,460,735.875 to 26,376,781.500)	197,238-356,609.942
Pediatric trauma (indicator variable)	0.052 (0.222)	0.000 (1 to 1)	0.000-1.000
Medical center (indicator variable)	0.112 (0.315)	0.000 (1 to 1)	0.000-1.000
Unemployment (%)	3.863 (1.222)	3.700 (3.100 to 4.400)	1.800-18.300
Poverty (%)	13.983 (5.183)	13.600 (10.300 to 16.800)	2.600-38.200

### Geospatial Results

**Figure 4:**
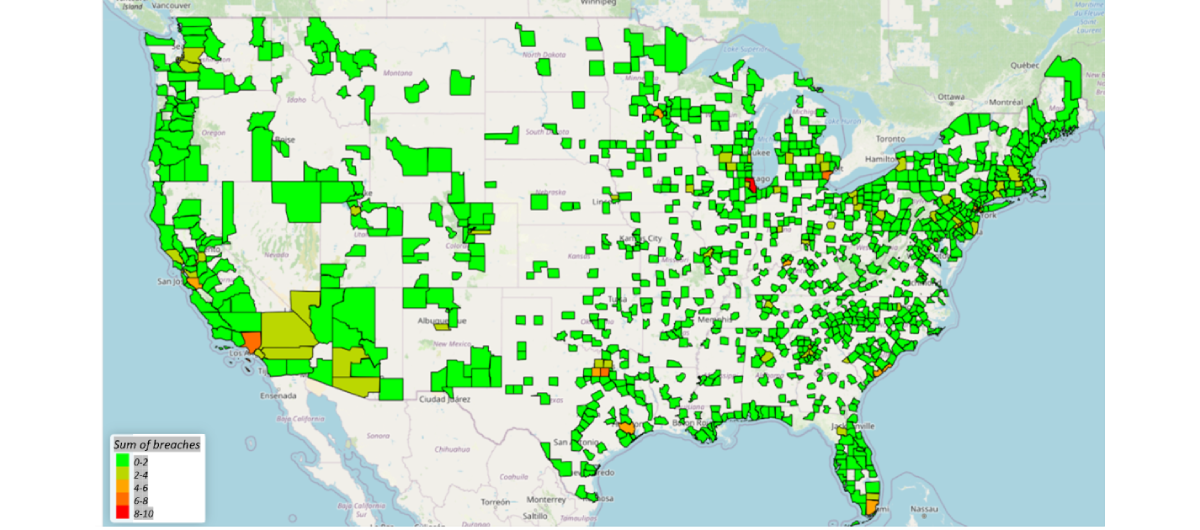
Sum of breaches by county.

**Figure 5:**
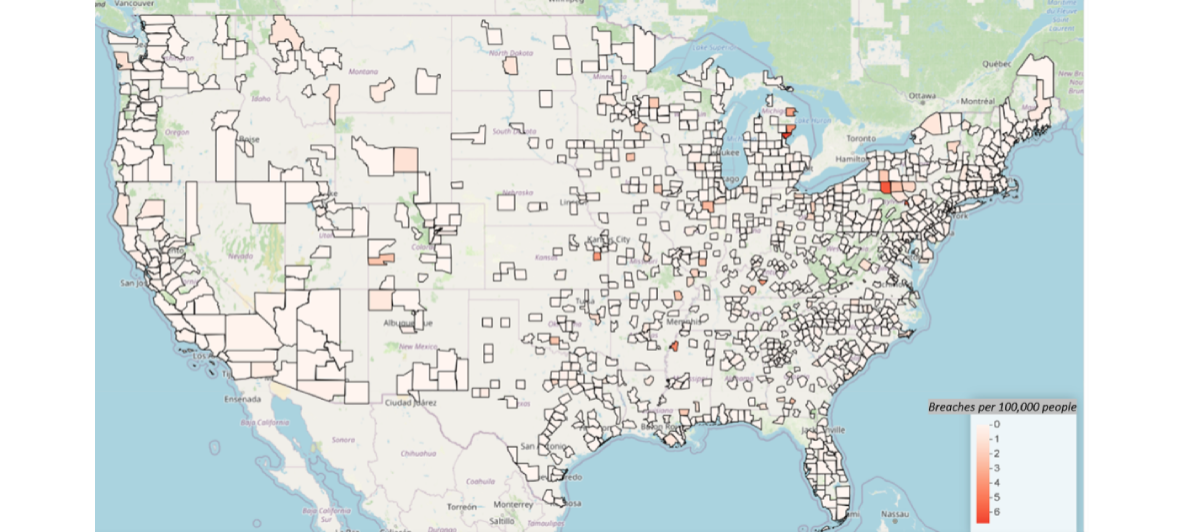
Breaches per 100,000 people by county.

Figures 4 and 5 provide maps of the counties with the sum of breaches (Figure 4) and the breach rate per 100,000 persons (Figure 5). In Figure 3, Cook County in Illinois along with New York County in New York saw the largest number of breaches (9), with Los Angeles County close behind with 7 breaches. The sum of breaches, however, is not population normalized as in Figure 5. In Figure 5, Arenac County, Michigan, has the highest number of breaches per 100,000 persons; however, the reason for that is that 1 breach was reported in a county that has <15,000 people. A web-based version of these maps is available on the web [41].

### Model 1: Logistic Regression

#### Overview

The full logistic regression model was run on the training set before predicting the test set along with L1 and L2 regularization. The L1 regularization resulted in no variables with 0 coefficients (eg, all variables retained in the model). The results for L2 also suggested that no variables should be removed. Thus, the focus of our analysis was on the full model and its submodels based on the variable groupings (eg, workload).

**Figure 6:**
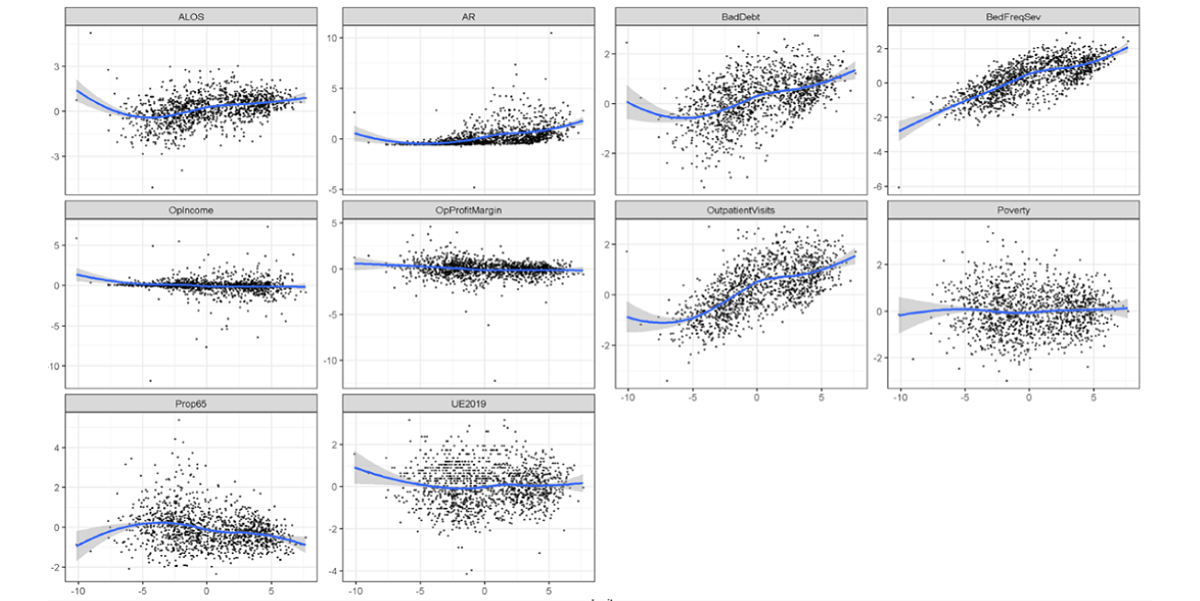
Linearity in the log odds for quantitative variables. Blue lines: locally estimated scatterplot smoothing (loess) curves. ALOS: average length of stay; AR: accounts receivable; BedFreqSev: bed frequency and severity; OPIncome: operating income; OPProfitMargin: operating profit margin; Poverty: poverty by county; Prop65: proportion of population over 65 years old; UE219: unemployment in 2019.

The full model’s pseudo-*R*^2^ was a moderate 0.576, and all assumptions were reasonably met except for the presence of outliers. The model exhibited nominal collinearity, with the highest VIF of 3.719 observed for the engineered inpatient workload variable. In addition, there were 6 potential extreme outliers identified through Cook’s distance assessment, using a threshold derived from the upper 0.5% of the distribution, given that no values exceeded 1.0. Visual inspection (Figure 6) indicated reasonable linearity in the logarithms for the quantitative variables. Moreover, there was a reasonable assumption of independence among observations [55].

**Figure 7:**
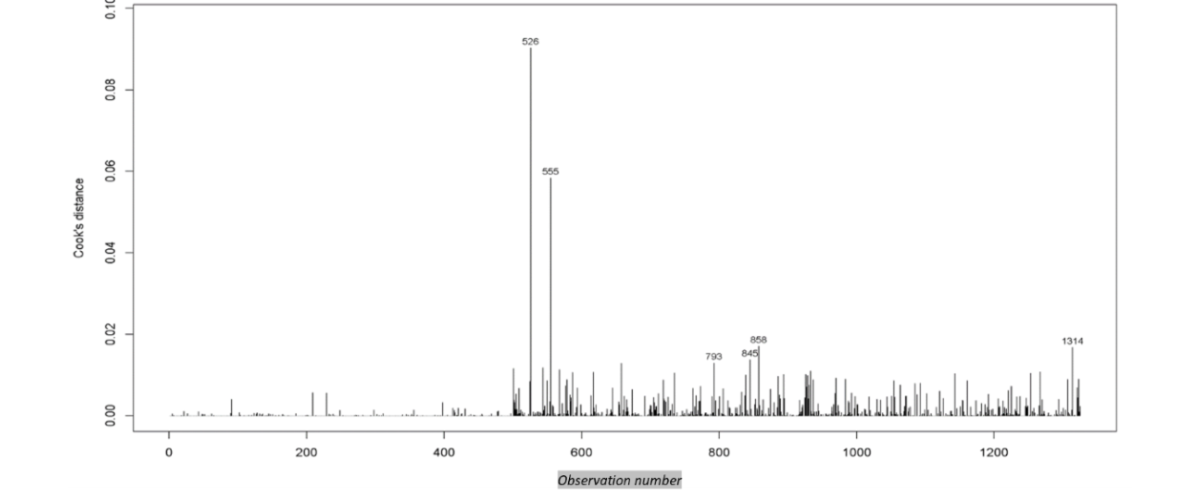
Plot of Cook’s distances with the top 6 extreme outliers noted.

Figure 7 depicts Cook’s distance by observation number. Cook’s distance measures the sum of squared differences between the estimated for the DV minus the estimate for the DV, excluding each observation separately, and divides that value by the rank of the independent variable matrix multiplied by the mean squared error. The resultant ratio is that of the 2 variances (error variance vs model variance) and can be measured as an *F* distribution. For large samples, this distribution will be near 1.0; therefore, a common recommendation is to remove the Cook’s distances >1. In this case, no distance exceeded 1.0; however, 6 were extreme statistical outliers. Removing these top 6 extreme outliers had no effect on the directionality of the estimated coefficients and nominal effects on any of the magnitudes of those coefficients; therefore, they were retained in the model. This comparison is available on the web. Furthermore, the prediction performance of the model did not improve with their removal. Table 2 provides the exponents of the coefficient estimates (ORs) along with the associated SEs, statistical significance, and VIFs.

As shown in Table 2, a total of 7 variables were not statistically significant in the model: the highest quantile for the Asian population proportion (Asian P100), the proportion aged >65 years, operating income, bad debt, outpatients, unemployment, and poverty. All other variables were associated with the presence or absence of breaches. ORs for the engineered inpatient workload frequency and severity variable were high (3.939), along with those associated with pediatric trauma (2.192; although only significant at the α=.10 level) and medical center status (2.596). Compared with their referent category (P20), American Indian, Black, and Hispanic populations had a reduced risk of breach.

**Table 2:** Odds ratios (OR) and other estimates for model 1, logistic regression^a^.

Variable	Values, OR (95% CI)	*P* value	Log odds (SE)	Variance inflation factor
American Indian P40	0.395 (0.237-0.659)	<.001	–0.928 (0.261)	1.391
American Indian P60	0.405 (0.222-0.737)	.003	–0.905 (0.306)	1.256
American Indian P80	0.254 (0.135-0.476)	<.001	–1.371 (0.321)	1.248
American Indian P100	0.325 (0.178-0.594)	<.001	–1.123 (0.307)	1.417
Asian P20	0.117 (0.055-0.251)	<.001	–2.142 (0.387)	1.323
Asian P40	0.273 (0.15-0.497)	<.001	–1.298 (0.306)	1.303
Asian P80	0.328 (0.185-0.579)	<.001	–1.116 (0.291)	1.547
Asian P100	0.757 (0.416-1.38)	.36	–0.278 (0.306)	2.064
Black P40	0.206 (0.114-0.37)	<.001	–1.581 (0.300)	1.418
Black P60	0.115 (0.062-0.213)	<.001	–2.162 (0.315)	1.626
Black P80	0.219 (0.118-0.407)	<.001	–1.518 (0.316)	1.931
Black P100	0.147 (0.072-0.3)	<.001	–1.92 (0.366)	2.425
Hispanic P40	0.423 (0.231-0.775)	.005	–0.861 (0.309)	1.434
Hispanic P60	0.274 (0.145-0.516)	<.001	–1.296 (0.324)	1.614
Hispanic P80	0.246 (0.135-0.446)	<.001	–1.404 (0.304)	1.772
Hispanic P100	0.104 (0.053-0.204)	<.001	–2.264 (0.344)	1.990
Pediatric trauma	2.192 (0.871-5.519)	.10	0.785 (0.471)	1.219
Medical center	2.596 (1.282-5.257)	.008	0.954 (0.360)	1.575
CapitalExp_P20	0.436 (0.199-0.956)	.03	–0.831 (0.401)	1.432
CapitalExp_P40	0.283 (0.145-0.554)	<.001	–1.262 (0.343)	1.259
CapitalExp_P80	0.54 (0.324-0.901)	.02	–0.616 (0.261)	1.460
CapitalExp_P100	0.507 (0.277-0.927)	.03	–0.679 (0.308)	1.988
Proportion aged >65 y	0.827 (0.652-1.048)	.12	–0.190 (0.121)	1.514
Operating profit margin	0.704 (0.558-0.889)	.003	–0.351 (0.119)	1.629
Operating income	1.156 (0.91-1.468)	.23	0.145 (0.122)	1.610
Accounts receivable	1.344 (1.042-1.735)	.02	0.296 (0.130)	1.672
Bad debt	1.105 (0.797-1.533)	.55	0.1 (0.167)	2.592
Inpatient workload	3.939 (2.595-5.98)	<.001	1.371 (0.213)	3.719
Outpatient visits	1.287 (0.922-1.795)	.14	0.252 (0.170)	2.470
Average length of stay	0.66 (0.494-0.882)	.005	–0.416 (0.148)	2.026
Unemployment in 2019	0.839 (0.66-1.068)	.16	0.175 (0.123)	1.732
Poverty	1.232 (0.957-1.587)	.11	0.209 (0.129)	1.800

^a^P20 through P100: the quantiles for each of the discretized variables.

#### Submodel Analysis

**Figure 8:**
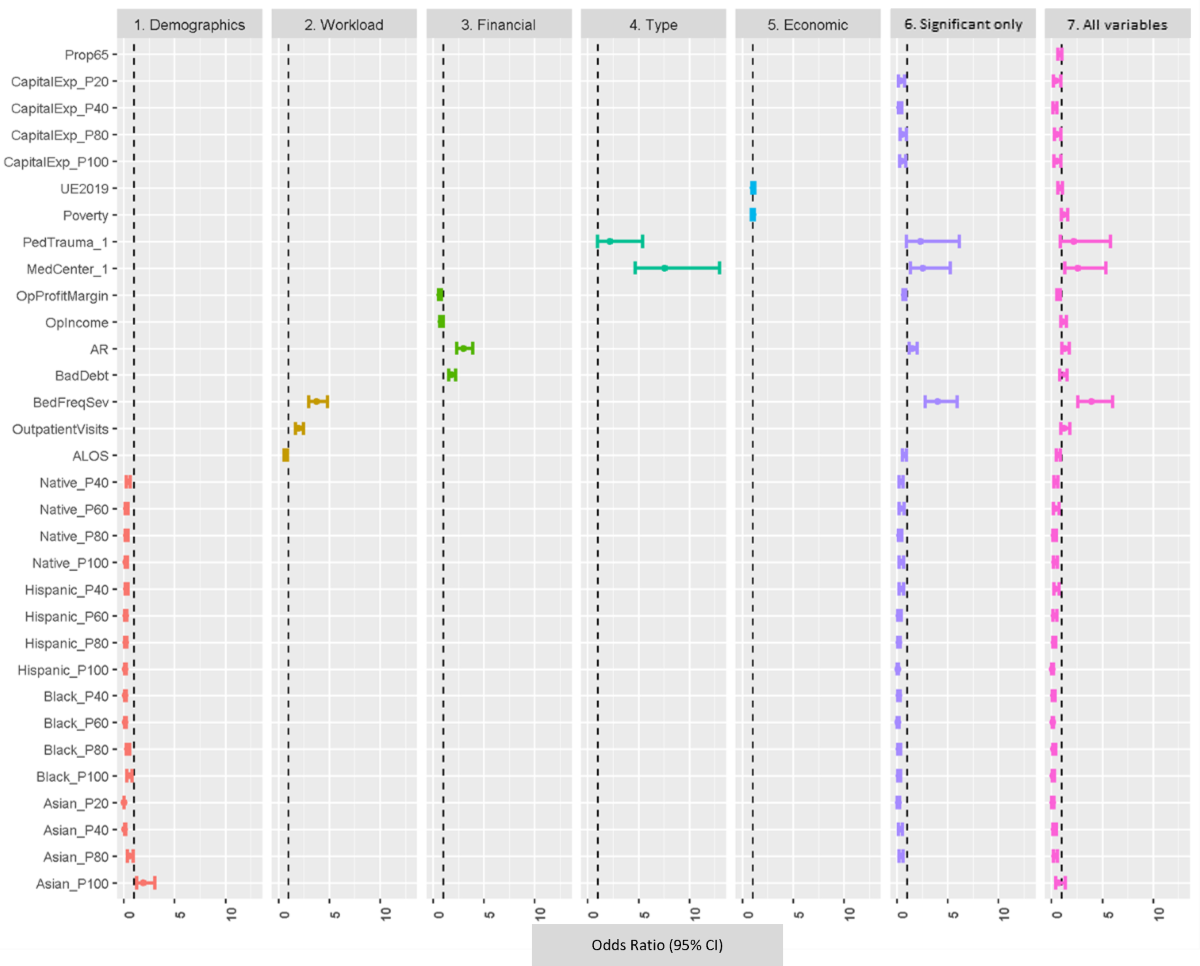
Forest plot of odds ratios for model 1 and submodels. P20 through P100: the quantiles for each of the discretized variables. ALOS: average length of stay; AR: accounts receivable; BedFreqSev: bed frequency and severity; CapitalExp: capital expenditures; MedCenter: medical center; Native: American Indian; OPIncome: operating income; OPProfitMargin: operating profit margin; PedTrauma: pediatric trauma; Poverty: poverty by county; Significant only: significant only model; UE219: unemployment in 2019.

Submodels by variable groupings were also evaluated along with a model containing only statistically significant variables (α=.10 level). Figure 8 shows the forest plots of all the submodels, the model with statistically significant variables, and the full model.

#### Model Performance on Test Set

The models estimated from the training set were then applied to the test set to estimate the classification metrics. The results for model 1 and the submodels are provided in Table 3.

In Table 3, the “Demographics” variables exhibit poor recall (0.375) and precision (0.353), resulting in a poor *F*1-score (0.364), the harmonic mean of precision and recall. “Workload” variables exhibit reasonable recall (0.833) but poor precision (0.430), indicating that nonbreaches are overclassified as breaches. The “Economics” subset has high recall but low positive predictive value (0.239) and specificity (0.157), indicating that it is overclassifying nonbreaches as breaches. The prediction accuracy of the subset “Type” is highest; however, its recall (sensitivity) is <0.5 (0.396) and thus fails to detect breaches well. The “Economics” variables are not credible classifiers, given accuracy (0.324), specificity (0.157), precision (0.230), and the *F*1-score (0.364). The “Finance” variables have the highest *F*1-score and reasonable metrics overall. This submodel performed better than the full model (model 1) on all metrics except for specificity (0.805 vs 0.862) and precision (0.523 vs 0.532). The “Full” model exhibits dominance over the model with the subset of statistically significant variables. Overall, the best 2 models are model 1 and the classifier with only financial variables.

**Table 3:** Test set performance metrics for model 1 and submodels.

Measure	Demographics-only model	Workload-only model	Finance-only model	Type-only model	Economics-only model	Model with subset of significant variables only	Full model
Accuracy	0.696	0.705	0.783	0.831	0.324	0.778	0.783
Recall (sensitivity)	0.375	0.833	0.708	0.396	0.875	0.521	0.521
Specificity	0.792	0.667	0.805	0.962	0.157	0.855	0.862
Precision (positive predictive value)	0.353	0.430	0.523	0.760	0.239	0.521	0.532
Negative predictive value	0.808	0.930	0.901	0.841	0.806	0.855	0.856
*F*1-score	0.364	0.567	0.602	0.521	0.375	0.521	0.526

### Model 2: Perceptron

Class weighting of the breach or no breach status was evaluated via hyperparameter tuning using only the training set data. The optimal weights estimated from the training data were {0: 0.8, 1: 0.2}. These weights were then applied and investigated on the test set. Regularization did not improve performance. The model 2 perceptron coefficients were directionally congruent with the logistic regression. The hyperparameter-tuned model 2 model achieved 79.7% accuracy, 54.2% recall, and 56.5% precision, with an *F*1-score of 0.553. The reason for this moderate performance was the selection of the aggregation link and the pursuit of a model that included only 1 neuron per independent variable. The model weights are presented in Table 4.

**Table 4:** Model coefficients’ performance metrics on the test set data^a^.

Variable	Perceptron model	Support vector machine model	Log odds (logistic regression)	Coefficient directionality
American Indian P40	−8.400	−0.665	−0.928	All negative
American Indian P60	−9.600	−0.576	−0.905	All negative
American Indian P80	−13.200	−0.911	−1.371	All negative
American Indian P100	−6.000	−0.749	−1.123	All negative
Asian P20	−16.800	−1.239	−2.142	All negative
Asian P40	−6.000	−0.844	−1.298	All negative
Asian P80	−10.200	−0.562	−1.116	All negative
Asian P100	−3.000	−0.170	−0.278	All negative
Black P40	−13.800	−0.829	−1.581	All negative
Black P60	−16.200	−1.152	−2.162	All negative
Black P80	−12.000	−0.824	−1.518	All negative
Black P100	−13.200	−1.003	−1.920	All negative
Hispanic P40	0.600	−0.497	−0.861	Mixed
Hispanic P60	−9.000	−0.638	−1.296	All negative
Hispanic P80	−13.800	−0.715	−1.404	All negative
Hispanic P100	−14.400	−1.301	−2.264	All negative
Pediatric trauma	7.200	0.755	0.785	All positive
Medical center	7.800	0.921	0.954	All positive
Capital expenditures P20	−5.400	−0.447	−0.831	All negative
Capital expenditures P40	−8.400	−0.680	−1.262	All negative
Capital expenditures P80	−4.200	−0.255	−0.616	All negative
Capital expenditures P100	−7.200	−0.307	−0.679	All negative
Proportion aged >65 y	−2.518	−0.087	−0.190	All negative
Operating profit margin	−1.435	−0.135	−0.351	All negative
Operating income	1.236	0.008	0.145	All positive
Accounts receivable	3.168	0.287	0.296	All positive
Bad debt	0.517	−0.024	0.100	Mixed
Bed frequency severity	15.404	0.723	1.371	All positive
Outpatient visits	0.056	0.118	0.252	All positive
Average length of stay	−1.336	−0.221	−0.416	All negative
Unemployment	−1.535	−0.039	−0.175	All negative
Poverty	0.194	0.196	0.209	All positive

^a^P20 through P100: the quantiles for each of the discretized variables.

### Model 3: SVM

The SVM loss function to be minimized was selected via hyperparameter tuning on the training set. The hinge loss performed better on the training set data and was thus carried forward. This function is expressed as the minimization of the following function: maximum (0,1 – ***ŷ*** X *y*). In this function, *y* is the DV on the set {−1, 1}, and ***ŷ*** is the classifier score. Thus, if the classifier score is 1 and the true observation is also 1, then the loss function is 0. However, any mismatch results in a loss equal to 2 [56]. Again, regularization did not improve the model performance. The results of the SVM classification on the test set were reasonable, scoring 83.1% accuracy, 65.1% precision, 58.3% recall, and the highest *F*1-score of all models (0.615). The coefficients were directionally congruent with the logistic regression and perceptron models. The model weights are shown in Table 4.

### Coefficient and Metric Comparisons

A comparison of the coefficient stability and model performance metrics was necessary to evaluate model validity. Table 2 provides the model coefficients for model 1 (logistic regression), model 2 (perceptron), and model 3 (SVM). Out of the 32 estimated coefficients (excluding the intercept), 30 (94%) were directionally congruent across all 3models; only the perceptron differed on 2 (6%) coefficients (Hispanic P40 and bad debt).

Table 5 shows the correlation coefficients for all 3 models. Correlations ranged from 0.934 to 0.982, indicating the directional congruence of the coefficients.

Table 6 presents a comparison of the metrics across the 3 models. In this table, the SVM shows dominance. However, all 3 models perform the classification task well with directional congruence of weights.

**Table 5:** Correlation matrix for the coefficients of all 3 models.

Models	Perceptron model	Support vector machine model	Logistic regression model
Perceptron model	1	0.934	0.954
Support vector machine model	0.934	1	0.982
Logistic regression model	0.954	0.982	1

**Table 6:** Accuracy metrics for model 1 (full) through model 3.

Measure	Perceptron model	Support vector machine model	Logistic regression model
Accuracy	0.797	0.831	0.783
Recall (sensitivity)	0.542	0.583	0.521
Specificity	0.874	0.906	0.862
Precision (positive predictive value)	0.565	0.651	0.532
Negative predictive value	0.863	0.878	0.856
*F*1-score (breaches)	0.553	0.615	0.526

## Discussion

### Principal Findings

First, the model coefficient performance metrics indicate convergent validity across models (perceptron, SVM, and logistic regression ) for all variables except bad debt and counties with Hispanic populations >20% and up to 40%, both of which had mixed results on coefficient directionality. Second, the SVM model was the dominant model across all the 3 class models. Third, all 3 models performed classification well with directional congruence of weights.

For the final logistic regression predictive model, we discuss the predictors with the top 5 and lowest 5 ORs. The top 5 ORs, indicating the highest odds of experiencing a data breach, occurred for inpatient workload, medical center, pediatric trauma center, AR, and outpatient visits, in high to low order. The bottom 5 ORs, indicating the lowest odds of experiencing a data breach, occurred for Black populations of >20% and ≤40%, >80% and ≤100%, and >40% but ≤60% as well as counties with ≤20% Asians or between 80% and 100% Hispanic individuals, in high to low order. Overall, our results are in line with those of other studies that determined that patient workload, facility type, and financial outcomes were associated with the likelihood of health care data breach occurrence [20,23,38].

The most significant predictor of health care data breach occurrence was inpatient workload. Inpatient workload was a feature-engineered measure composed of the product of severity (number of acute beds), bed percentage use, and average CMI. Hospitals with higher CMIs typically specialize in treating complex or surgical cases, catering to patients needing greater resources and longer durations of care. In congruence with our results, other studies found that increased numbers of intensive care beds, number of staffed hospital beds, performing more surgical operations, and having a neonatal intensive care unit were correlated with a greater likelihood of data breaches [22,25].

Counties with a higher number of medical centers or pediatric trauma centers had higher odds of experiencing data breaches. This is not surprising because most large hospitals and health centers are in urban settings where a larger proportion of claims data is processed. These larger centers may present more lucrative targets for cyberattackers than their rural peers. Furthermore, medical centers tend to have free clinics staffed by medical students who serve medically underserved populations, and these facilities may have weaker cybersecurity perimeters.

The results for the financial outcomes were both nuanced and compelling. On the one hand, we noted an increased likelihood of a breach across all models as AR and operating income increased. We interpret this to imply that that as volumes and profitability expand, the attractiveness of the hospital to bad actors and the criminally motivated also increases. This appears to be supportive of our findings related to academic medical centers and facilities serving pediatric patients, both of which accommodate large clinical volumes across all demographic groups. On the other hand, our findings reflect a negative association related to operating profit margin. Given some hospitals’ capacity to expand cash flows via nonoperating revenue sources (eg, donations, investments, facility rental, and retail operations), it would appear that additional margins permit some hospitals to procure the staff and infrastructure necessary to thwart breach attempts. This appears to be supported by our findings related to capital expenditure, where we observed a negative association related to all levels of expenditure in comparison with our referent modal group. We infer this to mean that at lower levels of capital expenditure, the hospital is financially constrained, and thus, it is likely not a lucrative target for hackers to exploit. Conversely, at higher expenditure levels, facilities are more capable of dedicating sufficient resources to counteract or interdict breach efforts.

Regarding demographics, hospitals in counties with higher populations of Blacks and Hispanics had a lower risk of having a data breach. It may be the case that these counties have facilities facing financial challenges, and thus, they are not seen as attractive targets by cyberattackers. Age >65 years was not a significant predictor of health care data breach occurrence. Although there is no comparable research on demographic predictors at the county level, Chua et al [38] determined that age was related to individuals’ security awareness and compliance with organizational security practices, with older individuals having better security behaviors. Conversely, other studies have correlated increased age with lower levels of health literacy, which contributes to difficulty following instructions for the secure use of patient appointment systems, EHRs, telehealth applications, and web-based pharmacy systems [57-61]. Relevant to cybersecurity, medical information has a higher selling price on the internet than other industry data, making secured health care connectivity essential [62]. More research is needed on the associations of race, ethnicity, age, and the likelihood of data breaches.

The geospatial results provided a descriptive analysis, including the sum of breaches and the breach rate per 100,000 people by county. Cook County in Illinois, New York County in New York, and Los Angeles County in California had the largest number of breaches. This is consistent with the results from other studies on geospatial effects on health care data breaches [39,40]. These highly dense metropolitan areas most likely have larger IT budgets to defend themselves against cyberattacks.

### Theoretical and Practical Implications

Health care data breaches are a rapidly increasing type of data breach. Nevertheless, there is limited research on hospital characteristics, economics, demography, and geographical factors as predictors of health care data breaches. Our findings provide a predictive model for health care data breaches that may assist health care managers in reducing the risk of data breaches.

The findings suggest that race and ethnicity are associated with the likelihood of a data breach, which signals a need for initiatives to reduce this disparity. Older individuals may require additional technological support. As financial conditions permit, medical facilities should consider hiring bilingual technological support staff. The staff could create materials, videos, and handouts for patients on security best practices for internet use that could be posted on their web page. Individuals dealing with the challenges of unemployment and low incomes need group classes with demonstrations on security best practices. Clinical staff may wish to conduct health literacy assessments during routine medical visits. Patients and their guests may need a short cybersecurity course before accessing the internet on guest networks.

If a hospital falls into the high-risk category identified in this study, increased risk management is suggested. They should consider hiring more IT security specialists. Of course, this requires adequate funding, which, for some hospitals in some areas, is in short supply and cannot be generated at the hospital level. This implies that a more aggressive and targeted policy approach is warranted to assist these hospitals in fending off data breach attempts. However, there are low-cost interventions that can be considered by all hospitals. For example, HIPAA provides a web-based security risk assessment tool that is helpful in evaluating a facility’s risk levels [63]. Quarterly use of this tool would help organizations prepare for their mandatory HIPAA risk assessment. In addition, the new Cybersecurity Framework Implementation Guide is a useful tool that outlines security best practices [64].

### Limitations

Our study had several limitations. First, this study is drawn from a single cross-section of data for the year 2022; thus, we cannot explore the changes in our predictive variables over time. Second, there might be additional factors affecting the relationships we are examining that are not accounted for in our study data set. For example, staff training, hospital budgets, and security expertise may be relevant in predicting the number of hospital data breaches. Third, owing to the sparsity of data for some counties, the data were aggregated for analysis at the county level. A data set with fewer missing variables may have provided additional insight by allowing the examination of our study variables at the hospital level. Fourth, other patient population demographic variables were not included in our study, which may need to be considered because of the intersection of cybersecurity in the consumer industry and that of the hospital. Fifth, although the sample size was adequate, a larger sample could have produced a model with more significant predictors and a better representation of data breaches by county across the United States. Sixth, this was a nonexperimental quantitative study that can show associations between predictors and criterion but cannot prove cause and effect between the predictors and the criterion.

### Recommendations for Future Work

Future research might consider the association between health care data breaches and specific organizational characteristics using longitudinal data with a more complete data set. Access to hospital-level data with fewer missing variables may have allowed for hospital-level breach analysis and inferential geospatial analysis. A data set with staff training, hospital budgets, and security expertise data may have produced additional insights for our predictive model.

Demographics, such as the average age of residents in each county, should be considered in future work because counties with more young adults may have higher odds of breaches owing to more prevalent risky security behaviors. The educational attainment and work experience of state residents can affect the number of data breaches.

Another area to be explored is the number of EHRs, EMRs, and CPOEs; their use levels; and the number of intensive care beds and neonatal intensive care units. For example, participants in a Health Information Exchange could provide multiple attack points for malicious hackers, which may increase breach risk. Real-time location service used extensively on the wireless network for mobile medical devices such as infusion pumps also poses risks [32]. Facilities that use bar coding or biometrics may have a lower risk of breaches. Older facilities may have more legacy computer systems that cannot be updated with the latest security features, which increases the risk of data breaches.

The number of full-time personnel versus part-time or contract personnel should be considered because part-time employees may not be knowledgeable about the facility’s security specifications or may be less motivated toward security compliance. Information technology software in use at health facilities should be examined because the increased connectivity provided by EHRs, telemedicine consultations, mobile device use, the use of personal devices as part of bring your own device programs, and emails to health care providers open the door for cyberhackers. The number of health care consumers or employees working off-site could be associated with breach risk. Future research should analyze different data sets to determine if the results on financial variables, population density, and CMIs are unique to the data set in this study.

### Conclusions

Health care data breaches have been a topic of interest for several years. To date, there remains a research gap in the predictors of health care data breaches. In this study, we explored the demography, geography, economics, and hospital characteristics of health care data breach risks to close the research gap. These findings should illuminate the predictors of health care data breaches to facilitate risk management and provide guidance for health care managers to reduce the risk of data breaches.

## Abbreviations

ALOS: average length of stay
AR: accounts receivable
CMI: case mix index
CPOE: Computerized Provider Order Entry
DV: dependent variable
EHR: electronic health record
EMR: electronic medical record
HHS: Department of Health and Human Services
HIPAA: Health Insurance Portability and Accountability Act
OCR: Office of Civil Rights
OR: odds ratio
SVM: support vector machine
VIF: variance inflation factor
